# Molecular mechanisms underlying seed enlargement mediated by seed coat development in autotetraploid castor bean

**DOI:** 10.1111/tpj.71146

**Published:** 2026-09-28

**Authors:** Dan He, Tianlong Yang, Xianxing Chen, Xueyong Xu, Chen Liu, Anmin Yu, Bing Han, Aizhong Liu, Jian Wu

**Affiliations:** ^1^ Key Laboratory for Forest Resources Conservation and Utilization in the Southwest Mountains of China Ministry of Education, Southwest Forestry University Kunming 650224 China; ^2^ Yunnan Provincial Key Laboratory for Conservation and Utilization of In‐Forest Resource Southwest Forestry University Kunming 650224 China; ^3^ Germplasm Bank of Wild Species Kunming Institute of Botany, Chinese Academy of Sciences Kunming 650201 China

**Keywords:** castor bean, autotetraploid, seed size, seed coat, cell expansion, lignin

## Abstract

Polyploids, particularly synthetic lines, often produce larger seeds than their diploid progenitors. However, little is known regarding the role of seed coat development in the larger seeds formation in polyploid plants. Here, we investigated the cellular and molecular basis by which seed coat development mediates seed enlargement in the oilseed crop castor bean (*Ricinus communis* L.) following homologous chromosome doubling. We found that seeds of autotetraploid castor bean, including seed length, width, thickness and hundred‐seed weight, were significantly greater than those of diploids. Histological analysis further revealed that the enlarged seeds were associated with increased seed coat cell area. At the transcriptome level, we showed that genome doubling led to stage‐specific and non‐linear transcriptional changes. A total of 6043 non‐redundant differentially expressed genes (DEGs) were identified between tetraploid and diploid seed coats. Among them, several key genes related to cell expansion and cell wall loosening (e.g. *RcEXPA1*/*5*/*9*/*12*/*15* and *RcEXLA1*, *RcEXTs*, *RcELP*, *RcCESA4/7/8*, *RcXYL1*, and *RcXTH15*/*27*/*30*) were significantly up‐regulated in tetraploids, consistent with enhanced seed coat expansion. In addition, lignin biosynthesis‐related genes (*RcPAL1*, *RcCOMT1*, *Rc4CL2*, *RcCSE*, *RcC4H*, *RcCCRs*, *RcCADs*, *RcCCoAOMT1*, and *RcHCT*) were significantly down‐regulated in tetraploids, which may facilitate seed coat cell enlargement. The stage‐specific DEGs in the indole‐3‐acetic acid (IAA), gibberellin, and brassinosteroid signaling pathways (*RcIAA3*/*13*/*16*/*26*/*27*, *RcRGL2*, and *RcBSK6*) may contribute to regulating seed coat cell development. Importantly, co‐expression analysis together with yeast one‐hybrid, electrophoretic mobility shift assay, and dual‐luciferase reporter (DLR) assays showed that RcPDF2 bound to two L1‐box elements in the *RcEXLA1* promoter and increased its promoter activity, suggesting that RcPDF2 contributes to seed size determination by modulating seed coat cell expansion. Collectively, our study provides important insights into the underlying molecular basis of gene dosage effects on seed size formation in autopolyploid plants, thereby laying a solid foundation for enhancing yield in castor bean through polyploid breeding.

## Significance Statement

The mechanism by which whole‐genome duplication promotes seed enlargement remains unclear, particularly the contribution of maternal seed coat tissues. This study reveals that seed coat cell expansion drives seed enlargement in autotetraploid castor bean and elucidates a transcriptional regulatory module in which RcPDF2 directly activates the expression of *RcEXLA1* to regulate seed coat cell expansion, thereby leading to seed enlargement in autotetraploids. These findings provide a mechanistic framework for polyploid‐induced yield enhancement in oilseed crops.

## INTRODUCTION

Polyploidy, or whole‐genome duplication (WGD), has long been considered a fundamental driver of evolutionary processes in angiosperms (Otto, 2007; Tamayo‐Ordóñez et al., 2016). This phenomenon results in an increased chromosome count through the multiplication of an entire genome. Polyploids are categorized based on the origin of their genome multiplication into two main types: allopolyploids, which arise from the combination of two or more distinct genomes, and autopolyploids, which originate from the duplication of a single genome (Corneillie et al., 2019). WGD is believed to enhance genomic flexibility, often manifested through the functional divergence of duplicated genes, changes in transcriptomic abundance, genomic or chromosomal rearrangements, and altered gene dosage effects (Comai, 2005). Allopolyploids have offered valuable insights into how genome doubling contributes to phenotypic variation. However, the interpretation of allopolyploid phenotypes is often complicated by the combined effects of both genome duplication and hybridization. By comparison, autopolyploids display only gene dosage effects resulting from the duplication of their genetic material (Chen, 2007; Parisod et al., 2010). Therefore, colchicine‐induced synthetic autopolyploids provide an ideal system for identifying the phenotypic consequences of WGD.

The doubling of homologous chromosomes causes a global increase in gene dosage in plants, which may disrupt the expression patterns of numerous genes and ultimately contribute to phenotypic variation (Anssour et al., 2009). Several studies have shown that polyploid plants often exhibit enhanced vegetative growth and higher crop yields compared to their diploid counterparts, making polyploidy a valuable approach in plant breeding (Tsukaya, 2013; Zhou et al., 2015). For instance, cell size increases with higher ploidy level, leading to significantly larger seeds in autotetraploid *Arabidopsis thaliana* (Leitch & Leitch, 2022). Autotetraploid watermelon produces significantly larger seeds than its diploid counterpart (Garcia‐Lozano et al., 2021). It is also found that autotetraploid *Raphanus sativus* L. developed markedly larger seeds than those of diploid counterparts (Cheng et al., 2019). Similar seed enlargement traits have been reported in multiple tetraploid crop and horticultural species, such as *Brassica rapa* (Harun et al., 2021), *Oryza sativa* (Zhang et al., 2015), and *Mangifera indica* (Barceló‐Anguiano et al., 2021). While accumulating evidence indicates that WGD affects seed size, the developmental processes responsible for seed enlargement and their regulatory mechanisms are still poorly understood.

Castor bean (*Ricinus communis* L.) is recognized as an economically valuable non‐edible oilseed crop, widely cultivated across several regions of the world, particularly in India, Brazil, and China (Qiu et al., 2010). Castor oil is extensively used in the production of lubricants, nylon, adhesives, aviation fuels, furniture coatings, and biodiesel (Ogunniyi, 2006). As seed size is a critical trait closely associated with crop yield in castor bean, understanding the molecular mechanisms that regulate seed size formation is essential for the development of genetically improved varieties. Recently, we successfully generated autotetraploid lines of castor bean through colchicine‐induced chromosome doubling of the diploid variety ZB306, and observed that these synthetic autotetraploids produced larger seeds with higher oil content. Subsequent transcriptome analysis revealed several key genes involved in the regulation of seed endosperm development and oil biosynthesis in autotetraploid castor bean (Han et al., 2025). Unfortunately, the underlying molecular basis governing seed enlargement in autotetraploid castor bean remains largely unclear. Previous research has indicated that seed size differences between large‐ and small‐seeded genotypes were mainly associated with increased seed coat cell number and delayed lignification rather than cell expansion (Yu et al., 2019). Nevertheless, little is known about the cellular and molecular basis of seed enlargement mediated by seed coat development in autotetraploid castor bean during seed development.

In this study, we investigated the cellular and molecular basis of seed enlargement mediated by seed coat development following whole‐genome doubling in castor bean. Using the diploid variety ZB306 and its synthetic autotetraploid counterpart, we characterized phenotypic changes in seed size and seed coat development, and identified key mechanisms underlying seed enlargement. Our findings elucidate the molecular basis of how seed coat development mediates seed size formation in autotetraploid castor bean, and provide important insights and genetic targets for improving traits in castor bean breeding programs.

## RESULTS

### Comparative analysis of seed traits between diploid and tetraploid castor bean

Seed size is an important agronomic trait that determines crop yield. To assess the impact of ploidy level on seed traits, we compared the phenotypic traits of mature seeds between diploids and tetraploids. Comparison between the two ploidy levels revealed clear phenotypic divergence, with tetraploid plants exhibiting significantly enlarged seeds (Figure 1A) compared to their diploid counterparts. Seed length, width, thickness, and hundred‐seed weight increased by 4.19, 12.41, 11.88, and 3.01%, respectively, in tetraploids (Figure 1B–E). These results demonstrate that tetraploid castor bean produces enlarged seeds, consistent with previous reports in other tetraploid species such as watermelon, radish, and rice (Cheng et al., 2019; Garcia‐Lozano et al., 2021; Zhang et al., 2015).

**Figure 1:**
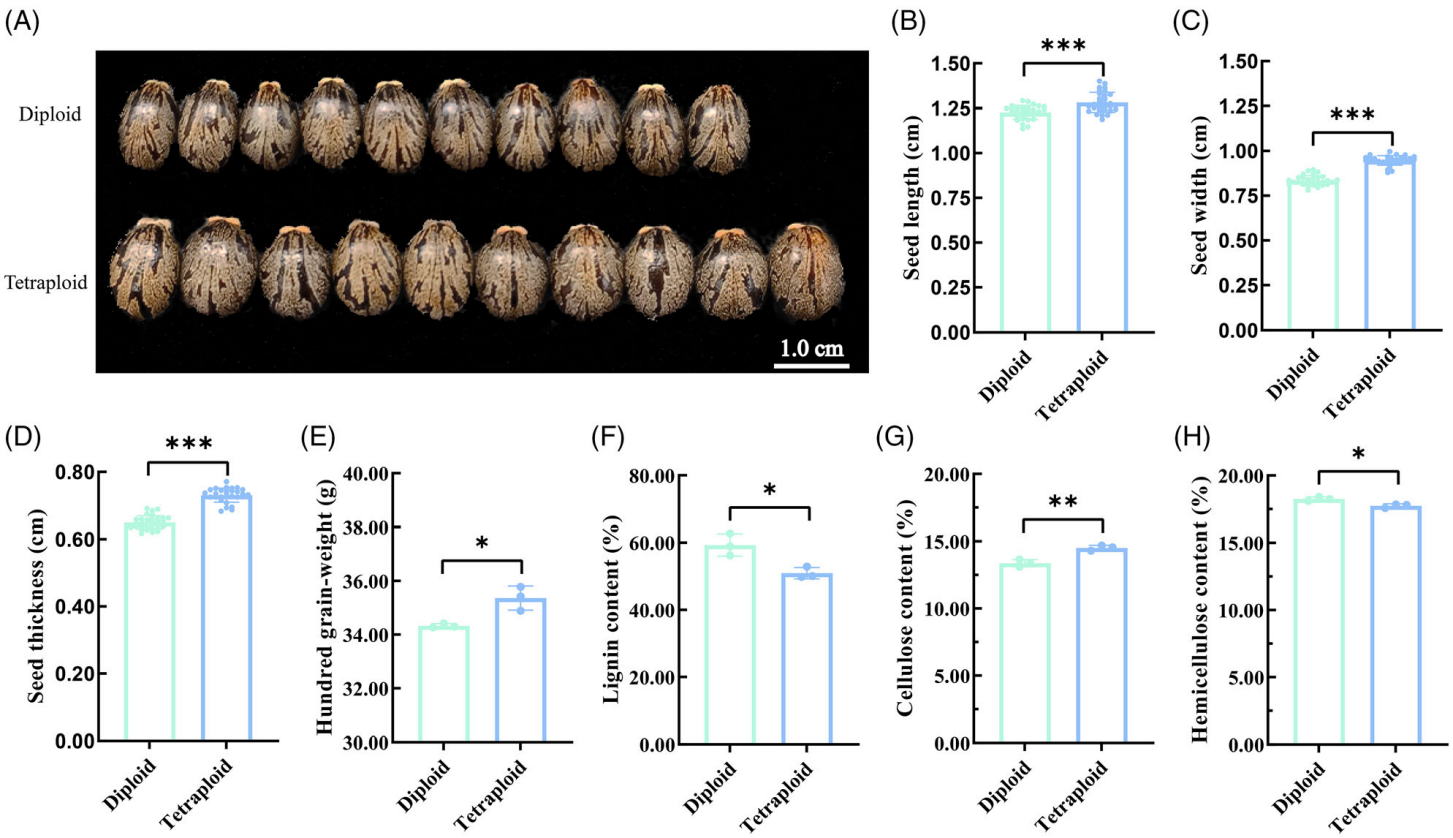
Changes of seed sizes in diploid and tetraploid castor beans. (A) Observed differences in seed size between diploids and tetraploids. Statistical analyses of seed length (B), width (C), thickness (D), hundred‐ seed weight (E), lignin content (F), cellulose content (G), and hemicellulose content (H) in mature seed coats of diploids and tetraploids. Data are shown as means ± SD of three independent biological replicates. Asterisks indicate significant differences between diploids and tetraploids determined using Student's *t*‐test (*0.01 < *P* < 0.05, **0.001 < *P* < 0.01, ***P < 0.001).

Lignin deposition in the cell wall generally enhances the mechanical strength of the seed coat, yet limits seed volume expansion and thereby affects seed size in castor bean (Yu et al., 2019). To investigate the effects of cell wall component content differences on seed size in tetraploid castor bean, we determined the contents of lignin, cellulose, and hemicellulose in the seed coats. The results showed that lignin and hemicellulose contents in tetraploid seed coats were significantly lower than those in diploids, whereas cellulose content in tetraploid seed coats was significantly higher than that in diploids (Figure 1F–H), suggesting that reduced lignin deposition may contribute to the increased seed size observed in tetraploids.

To elucidate the cellular mechanism underlying seed enlargement in tetraploids, we examined seed size and the cross‐sectional characteristics of seed coat cells in diploids and tetraploids at 10, 35, and 55 days after pollination (DAP). Consistent with a ploidy‐driven growth pattern, tetraploid seeds were larger than their diploid counterparts at all stages (Figure 2A). Histological examination revealed that, regardless of the stage, seed coat cell sizes in tetraploids were notably larger than those in diploids (Figure 2B). In particular, the late developmental stage was marked by both a denser packing of cells and a substantial increase in individual cell dimensions in tetraploids (Figure 2C). In contrast, a significantly higher cell number was observed only in tetraploids at 10 DAP, but this disparity gradually diminished in subsequent stages (Figure 2D). Seed coat thickness also showed a persistent increase in tetraploids relative to diploids throughout developmental stages (Figure 2E). Collectively, these observations demonstrate that cell enlargement, rather than persistent cell proliferation, serves as the primary cellular driver of increased seed size in tetraploids. This aligns with the well‐documented effects of ploidy elevation in plants (Li et al., 2012).

**Figure 2:**
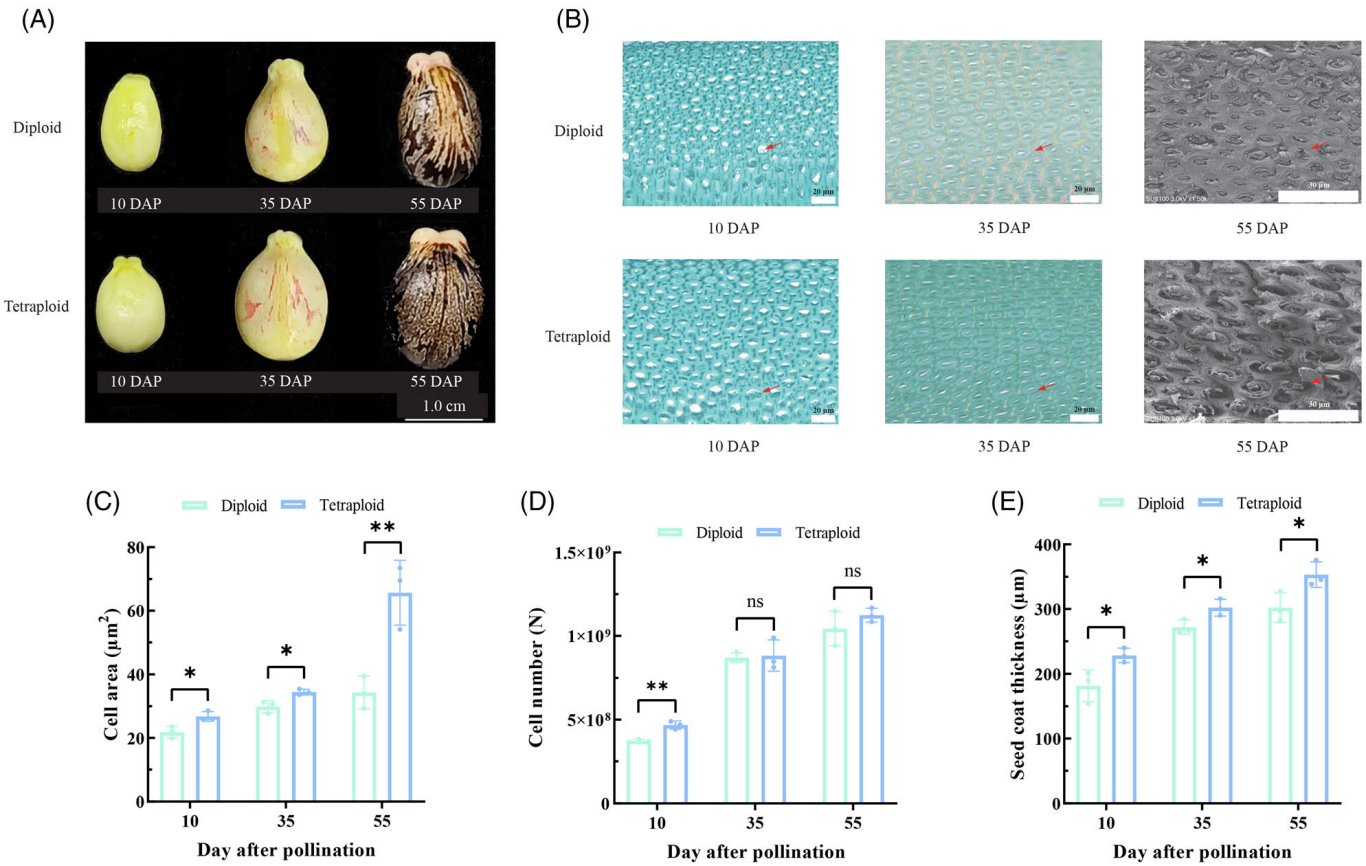
Characteristics of seed size changes at three development stages in diploids and tetraploids. (A) Enlarged seed size in diploids and tetraploids at three developmental stages. (B) Enlarged seed coat cells in diploids and tetraploids at three developmental stages. Statistical analysis of seed coat cell size (C), cell number (D), and seed coat thickness (E) in diploids and tetraploids at three developmental stages. SC, seed coat. Asterisks mark significant differences between diploids and tetraploids determined using Student's *t*‐test (*0.01 < *P* < 0.05, **0.001 < *P* < 0.01).

To assess how ploidy influences plant hormone profiles, we measured the contents of indole‐3‐acetic acid (IAA), gibberellin (GA), brassinosteroid (BR), and cytokinin (CTK) in diploid and tetraploid seed coats at three developmental stages (Figure S1). Overall, tetraploids exhibited significantly higher content of IAA, GA, and BR compared to diploids. In contrast, no significant difference in CTK content was detected between the two ploidy levels. Notably, IAA content remained elevated in tetraploids, reaching approximately 1.65‐ and 1.69‐fold that of diploids at 10 and 35 DAP, respectively. Likewise, GA and BR contents were also increased in tetraploids, and these plant hormones likely act cooperatively with IAA to facilitate cell wall loosening and expansion. Together, these data indicate that coordinated up‐regulation of specific growth‐promoting plant hormones underlies seed coat enlargement in autotetraploid castor bean.

Integrated with our earlier observations, the enlarged seed phenotype in autotetraploid castor bean appears to result from a synergistic interplay between enhanced cellular expansion and diminished lignification in the seed coat, thereby alleviating physical constraints on seed growth.

### Comparative transcriptomic analysis between diploids and tetraploids

To investigate the transcriptional basis of seed coat mediated seed enlargement in tetraploid castor bean, RNA sequencing was performed on 18 seed coat samples from diploid and autotetraploid plants at 10, 35, and 55 DAP. In total, 143.74 Gb of clean reads were obtained. The clean data showed Q20, Q30, and GC contents above 98.40, 95.82, and 41.66%, respectively, confirming the high quality of the RNA‐seq. The clean reads were aligned to the *R. communis* cv. ZB306 reference genome and corresponding annotation obtained from EupDB (http://eupdb.liu‐lab.com/; Liu et al., 2024), with mapping rates ranging from 89.90 to 98.29% (Table S2). For independent validation of the RNA‐seq data, a total of nine differentially expressed genes (DEGs) were randomly selected for quantitative reverse transcriptase‐polymerase chain reaction (qRT‐PCR; Figure S2). The qRT‐PCR results were consistent with the RNA‐seq analysis, as the relative expression patterns of all tested genes matched those obtained from sequencing, thereby confirming the reliability of the transcriptome dataset.

To compare global transcriptional patterns between diploid and autotetraploid seed coats, we summarized the numbers of expressed genes and transcripts at the three developmental stages. As shown in Table 1, the transcript/gene ratios were similar between the two ploidy levels at 10 DAP (1.41 in diploids and 1.44 in tetraploids) and at 35 DAP (1.42 and 1.41, respectively). By contrast, at 55 DAP the ratio was higher in tetraploids than in diploids (1.41 and 1.27, respectively). These results suggest that the transcriptional effects of genome doubling were not uniform throughout seed coat development, but instead showed clear stage‐specific variation.

**Table 1:** Numbers of expressed genes and transcripts in diploid and tetraploid seed coats at different developmental stages

Ploidy	Genes	Transcripts	Transcripts per expressed gene
D1	17 270	24 415	1.41
T1	17 366	24 957	1.44
D3	17 624	24 939	1.42
T3	17 152	24 173	1.41
D5	16 136	20 527	1.27
T5	16 677	23 483	1.41

*Note*: D1, D3, and D5 represent diploid seed coats at 10, 35, and 55 DAP, and T1, T3, and T5 represent the corresponding tetraploid samples.

Analysis of gene expression distribution showed that most genes in all samples were lowly expressed (FPKM <1), and the proportion of these genes increased markedly at 55 DAP, particularly in diploids (Figure S3a). Genes with FPKM ≥50 represented a relatively small fraction of the transcriptome, and were slightly more frequent in tetraploids than in diploids. Further analysis of gene level dosage response showed that most genes deviated from a strict 1:1 scaling relationship after genome doubling. Only 6.1, 10.1, and 10.0% of genes exhibited a 1:1 dosage effect at 10, 35, and 55 DAP, respectively (Figure 3A). Genes with a >1:1 dosage response increased gradually from 4.5% at 10 DAP to 25.8% at 55 DAP, becoming the largest class at the late stage. In contrast, the combined proportion of genes showing dosage compensation and partial dosage compensation was greatest at 10 DAP (82.2%) and then decreased over development. The proportion of genes with a negative dosage effect peaked at 55 DAP (25.3%) (Tables S3–S5), coinciding with the largest difference in transcripts per expressed gene between diploid and autotetraploid seed coats (Table 1). To investigate the functional characteristics of genes showing relatively strong positive dosage responses during early seed coat development, genes exhibiting 1:1 and >1:1 dosage effects at 10 DAP were combined for Kyoto Encyclopedia of Genes and Genomes (KEGG) pathway analysis. These genes were mainly associated with motor proteins, endocytosis, amino sugar and nucleotide sugar metabolism, nucleotide sugar biosynthesis, starch and sucrose metabolism, MAPK signaling, and plant hormone signal transduction (Figure 3B; Table S6). By contrast, genes exhibiting negative dosage effects at 35 DAP were enriched in carbon metabolism, phenylpropanoid and flavonoid biosynthesis, phenylalanine metabolism, aromatic amino acid biosynthesis, and plant hormone signal transduction (Figure 3C; Table S7).

**Figure 3:**
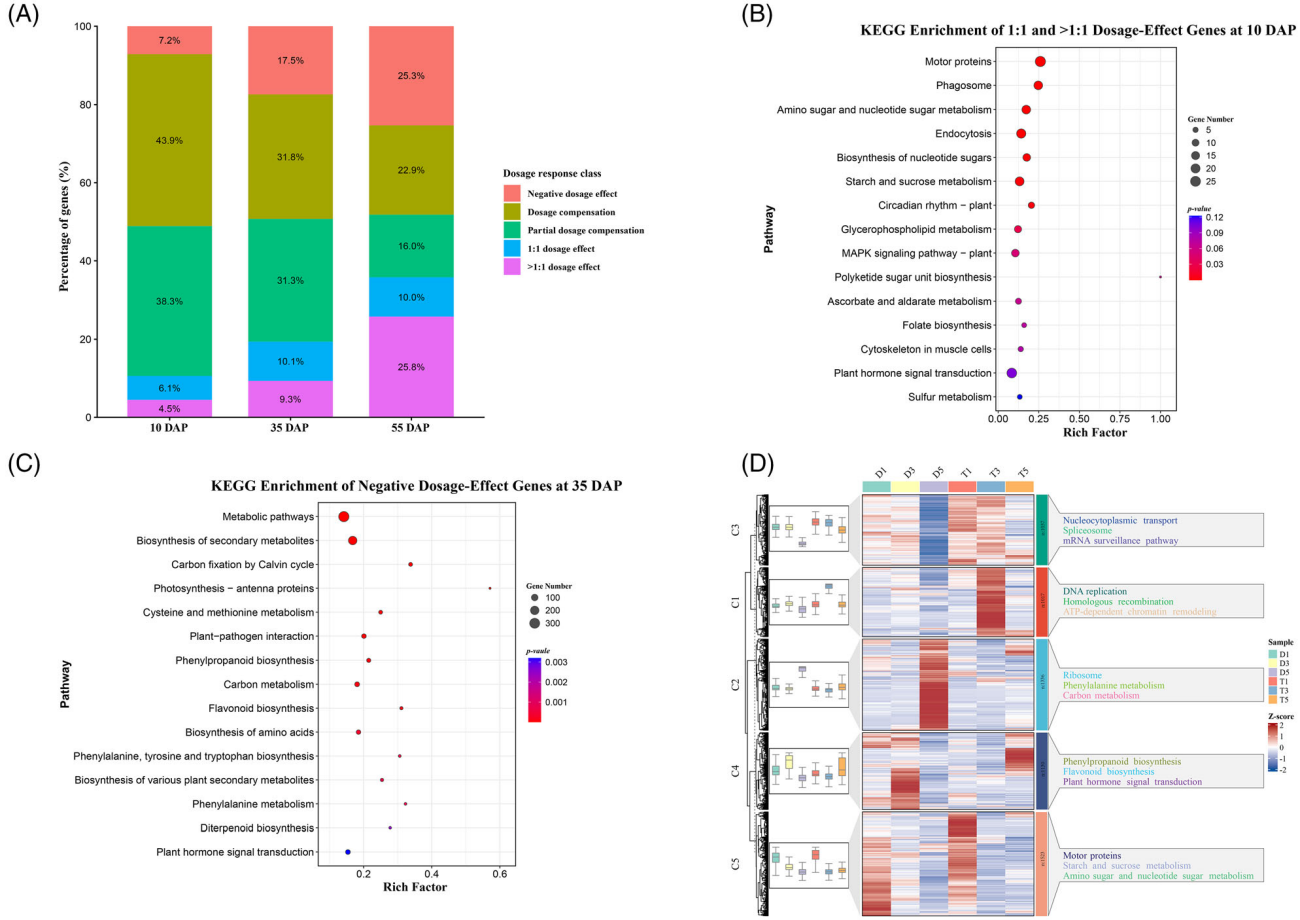
Gene dosage responses and differential expression patterns during seed coat development in diploid and autotetraploid castor bean. (A) Distribution of five dosage‐response classes among genes meeting the predefined expression threshold in diploid and tetraploid seed coats at 10, 35, and 55 days after pollination (DAP). (B) Kyoto Encyclopedia of Genes and Genomes (KEGG) enrichment analysis of genes showing 1:1 and >1:1 dosage responses at 10 DAP. (C) KEGG enrichment analysis of genes showing negative dosage responses at 35 DAP. (D) Expression pattern clustering and functional annotation of 6043 non‐redundant DEGs identified between diploid and tetraploid seed coats across the three developmental stages.

To further investigate transcriptional differences between diploid and tetraploid seed coats, DEGs were identified at the three developmental stages using DESeq2 software (Tables S8–S10). A total of 1029 DEGs were identified at 10 DAP (517 up‐regulated and 512 down‐regulated), 2634 at 35 DAP (981 up‐regulated and 1653 down‐regulated), and 3325 at 55 DAP (1504 up‐regulated and 1821 down‐regulated). Thus, the total number of DEGs increased as seed coat development progressed, while down‐regulated genes predominated at both 35 and 55 DAP, with the highest proportion of down‐regulated DEGs observed at 35 DAP (Figure S3b). The 6043 non‐redundant DEGs were then clustered based on their expression patterns across the six groups and divided into five clusters containing 1130, 1523, 1336, 1017, and 1037 genes, respectively (Figure 3D; Table S11). Cluster 1 was enriched in DNA replication, homologous recombination, and ATP‐dependent chromatin remodeling; Cluster 2 in ribosome function, phenylalanine metabolism, and carbon metabolism; Cluster 3 in nucleocytoplasmic transport, spliceosome, and mRNA surveillance; Cluster 4 in phenylpropanoid biosynthesis, flavonoid biosynthesis, and plant hormone signal transduction; and Cluster 5 in motor proteins, starch and sucrose metabolism, and amino sugar and nucleotide sugar metabolism. The five clusters showed distinct expression patterns across developmental stages and ploidy levels.

### Identification of DEGs involved in seed coat development

Seed coat development is a dynamic process involving coordinated cell division and cell expansion. In this study, we found that the cell size in tetraploid seed coats was significantly larger than those in diploids. Expansins, which mediate cell wall relaxation, are widely recognized as important regulators of cell enlargement. We identified 10 expansin‐related DEGs showing significant expression differences between tetraploid and diploid seed coats, most of which were up‐regulated in tetraploids. Especially, the expression of three expansin genes (*RcEXPA1*, *RcEXPA15*, *RcEXLA1*) was significantly up‐regulated in tetraploids at 10 and 35 DAP, indicating they likely play important roles during early seed coat cell development (Figure 4A; Table S12). Additionally, five extensin (EXT) or extensin‐like DEGs, *RcEXT3*, *RcELP1*, *RcELP4*, *RcELP5*, and *RcELP6*, were identified. *RcELP5* was significantly up‐regulated in tetraploids at 35 DAP. At 55 DAP, *RcEXT3* and *RcELP4* were significantly up‐regulated, whereas *RcELP1* and *RcELP6* were down‐regulated, indicating that these genes may be involved in middle‐ and late‐stage seed coat cell development. Six DEGs associated with cell division and the cell cycle were identified. In tetraploids, *RcCYCD5;1* and *RcCYCD6;1* were up‐regulated at 10 DAP, *RcCYCD3;2* was up‐regulated at 35 DAP, and *RcCYCD3;4* was down‐regulated at 35 DAP.

**Figure 4:**
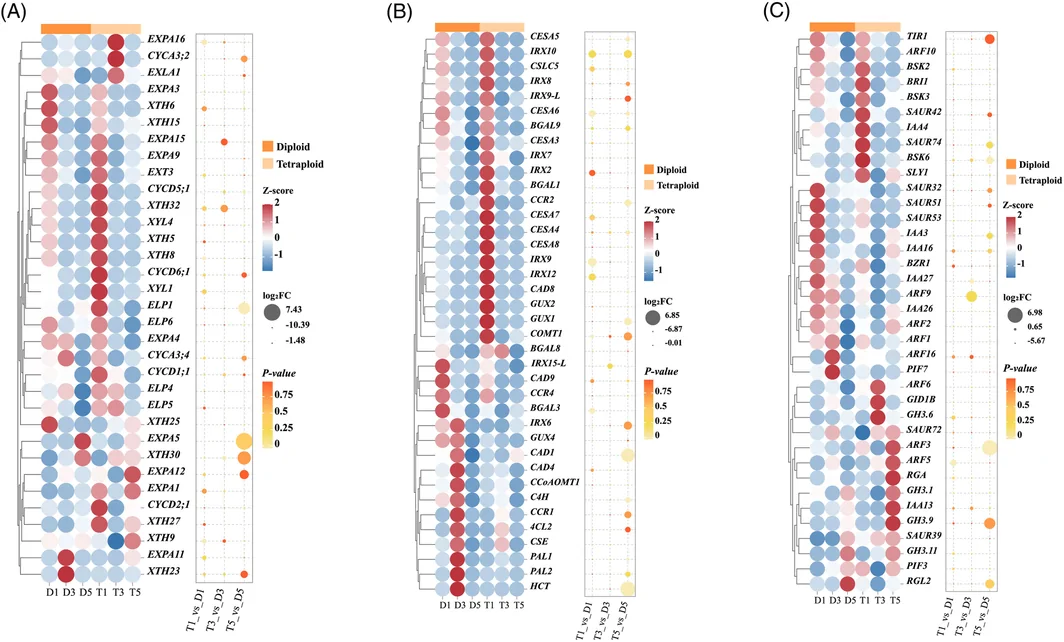
Expression profiles of genes associated with seed coat cell development in diploid and autotetraploid castor bean. (A) Expression profiles of genes involved in cell division, cell expansion, and cell wall remodeling. (B) Expression profiles of genes associated with cellulose, hemicellulose, and lignin biosynthesis. (C) Expression profiles of genes associated with IAA, gibberellin (GA), and brassinosteroid (BR) signaling pathway. In the adjacent dot plots, dot size represents the magnitude of log_2_FC in tetraploid relative to diploid seed coats, and dot color represents the corresponding *P* value.

Plant cell walls must maintain mechanical strength while remaining sufficiently extensible to allow cell growth. To investigate the molecular basis of this extensibility in tetraploid seed coats, we analyzed DEGs encoding enzymes associated with secondary cell wall (SCW) biosynthesis and remodeling. In total, 50 genes associated with SCW biosynthesis were examined (Figure 4A,B; Tables S12 and S13). Within the cellulose and hemicellulose biosynthesis pathways, eight genes (including *RcCESA4/7/8*, *RcIRX2/9*, *RcGUX1/2*, and *RcBGAL1*) were significantly up‐regulated in tetraploids at 10 DAP, whereas *RcIRX6* and *RcGUX4* were down‐regulated. At 35 DAP, *RcBGAL8* was significantly up‐regulated, while 12 genes (e.g. *RcCESA4/5/6/7*, *RcIRX6/8/9/10*, *RcGUX4*, *RcBGAL3/9*, and *RcCSLC5*) were significantly down‐regulated in tetraploids. At 55 DAP, *RcCESA3/5/6*, *RcIRX2*, and *RcBGAL1* were up‐regulated, whereas *RcIRX8*, *RcIRX15‐L*, and *RcBGAL8* were down‐regulated in tetraploids. Among genes associated with cell wall loosening and remodeling, *RcXTH27*, *RcXTH30*, and *RcXYL1* were significantly up‐regulated in tetraploids at 10 DAP, whereas *RcXTH6* was down‐regulated. At 35 DAP, *RcXTH6* and *RcXTH23* were significantly down‐regulated. At 55 DAP, *RcXTH15*, *RcXTH25*, and *RcXYL4* were up‐regulated, whereas *RcXTH30* was down‐regulated. In the lignin biosynthesis pathway, 15 DEGs encoding key enzymes showed significant expression differences between diploid and tetraploid seed coats. Most of these genes were consistently expressed at higher levels in diploids than in tetraploids across developmental stages. Notably, nine genes (including *RcPAL1*, *RcC4H*, *RcHCT*, *RcCOMT1*, *RcCCoAOMT1*, *RcCCR1*, *RcCAD1*, *RcCAD4*, *and RcCAD9*) were significantly down‐regulated in tetraploids at 35 DAP. This expression pattern is consistent with the lower lignin content observed in tetraploid seed coats. Collectively, these transcriptional changes in SCW biosynthesis pathways likely contribute to altered cell wall properties and enhanced cell expansion in tetraploid castor bean seed coats.

To clarify the involvement of plant hormones signaling in seed coat development, 37 DEGs associated with plant hormone signaling pathways were identified (Table S14), heatmaps were generated based on the FPKM values of genes (Figure 4C). In the IAA pathway, *RcARF16* and *RcSAUR74* were up‐regulated in tetraploid seed coats at 10 DAP, whereas *RcIAA16* and *RcSAUR72* were down‐regulated. At 35 DAP, five AUX/IAA transcriptional repressors (*RcIAA3/13/16/26/27*) and five ARF genes (*RcARF1/2/3/9/16*) were down‐regulated, whereas *RcGH3.6* and *RcGH3.11* were up‐regulated and *RcGH3.9* was down‐regulated. In addition, *RcSAUR32* and *RcSAUR53* were down‐regulated. At 55 DAP, RcTIR1 and four ARF genes (RcARF2/5/10/16) were up‐regulated, whereas several SAUR genes, including RcSAUR32/51/53/74, were down‐regulated. In the BR pathway, RcBSK6 was up‐regulated at 10 DAP, whereas RcBSK2, RcBSK3, and RcBZR1 were down‐regulated at 35 DAP. In the GA pathway, *RcGID1B* was up‐regulated at 35 DAP, whereas *RcRGL2* and *RcPIF7* were down‐regulated. At 55 DAP, *RcRGA* and *RcPIF7* were up‐regulated, while *RcRGL2* remained down‐regulated. Collectively, these hormone pathway‐associated DEGs exhibited stage‐specific expression patterns during seed coat development, suggesting that coordinated changes in IAA, BR, and GA signaling may contribute to the regulation of seed coat development and seed enlargement in autotetraploid castor bean.

### Analysis of transcription factors and gene co‐expression network

Transcription factors (TFs) are important regulators of seed development. To explore their involvement in seed coat cell development, we identified putative TFs from the castor bean protein used PlantTFDB. In total, 876 TF genes were identified, and the 20 most abundant TF families were shown in Figure 5A. MYB was the largest family (94 genes, 10.7%), followed by ERF (68, 7.7%), NAC (67, 7.6%), bHLH (59, 6.7%), WRKY (51, 5.8%), C2H2 (43, 4.9%), bZIP (36, 4.1%), GRAS (31, 3.5%), HD‐ZIP (30, 3.4%), and LBD (27, 3.1%). Among the 876 predicted TFs, 453 were annotated based on their best matches to Arabidopsis proteins and used for Mfuzz clustering (Figure 5B; Table S15). Five major expression clusters were obtained. Cluster 3 (*n* = 120) showed high expression at 10 DAP, whereas Cluster 4 (*n* = 91) showed relatively low expression in tetraploid seed coats at 35 DAP. Because the expression patterns of TF Clusters 3 and 4 were consistent with those of seed coat development‐related DEG Clusters 5 and 4, respectively, in Figure 3D, the 211 TFs in these two clusters were selected for subsequent co‐expression analysis.

**Figure 5:**
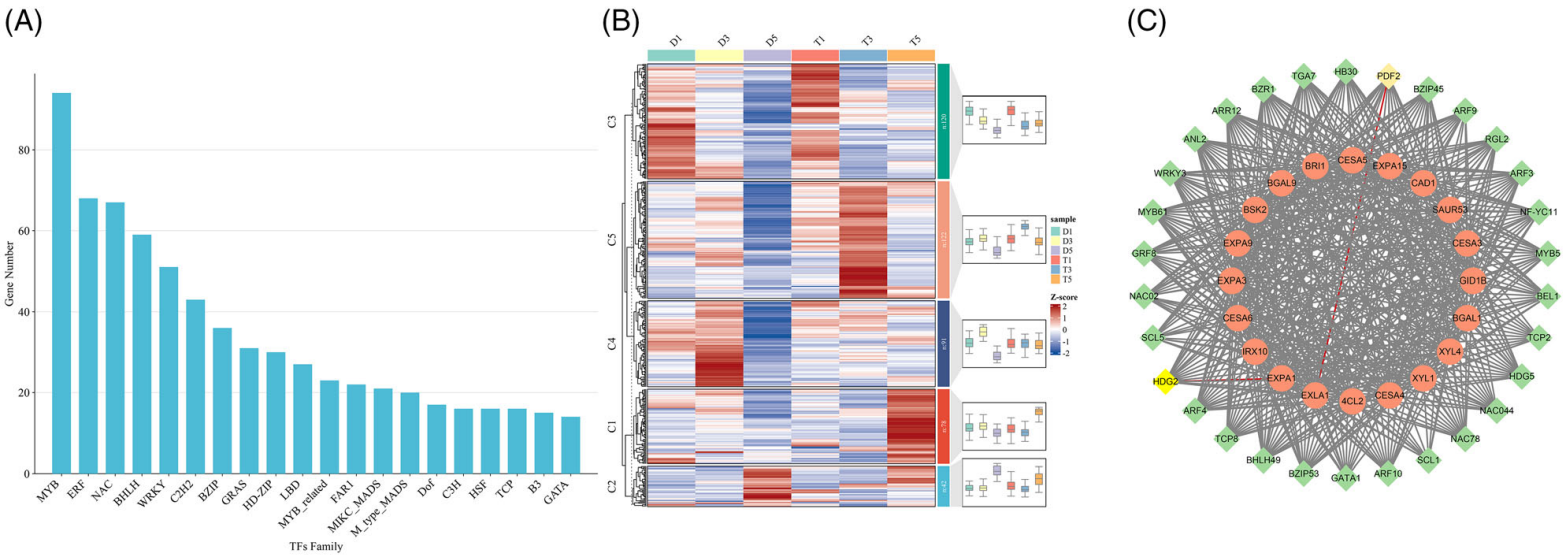
Identification and co‐expression analysis of transcription factors (TFs) associated with seed coat development. (A) Distribution of the 20 most abundant TF families among the 876 TFs identified in castor bean. (B) Mfuzz clustering of 453 annotated TFs based on their expression profiles in diploid and autotetraploid seed coats at 10, 35, and 55 days after pollination (DAP). Mean FPKM values from three biological replicates were *Z*‐score normalized. (C) Co‐expression network containing the 30 TFs and 20 seed coat development‐related genes with the highest node degrees. Diamonds represent TFs and circles represent seed coat development‐related genes. RcPDF2 and RcHDG2 are highlighted in yellow, other TFs in green, and genes in orange. *D* and *T* represent diploid and autotetraploid seed coats; D1/T1, D3/T3, and D5/T5 represent 10, 35, and 55 DAP, respectively.

To identify potential key regulators of seed coat development, we generated a co‐expression network consisting of 211 TFs in Clusters 3 and 4 and representative genes related to cell expansion, plant hormone signaling, cellulose and hemicellulose metabolism, and lignin biosynthesis. Pearson correlation analysis was performed across the 18 RNA‐seq samples, and correlations with |*r*| ≥ 0.8 and *P* < 0.05 were retained. Based on node degree, the 20 genes and 30 TFs with the highest connectivity were selected for the final network (Table S16). Notably, *RcEXPA1* and *RcEXLA1* showed the highest connectivity among the gene nodes. Promoter analysis identified two L1‐box elements in the *RcEXLA1* promoter and an HD‐ZIP‐binding element in the *RcEXPA1* promoter. Based on these co‐expression relationships and promoter *cis*‐element predictions, the RcPDF2‐*RcEXLA1* and RcHDG2‐*RcEXPA1* pairs were selected for experimental validation.

### Experimental validation of the RcPDF2‐ *RcEXLA1* and RcHDG2‐ *RcEXPA1* relationships

To test the two candidate regulatory relationships identified from the co‐expression network, yeast one‐hybrid (Y1H) and dual‐luciferase reporter (DLR) assays were first performed. Two L1‐box elements were identified in the tested *RcEXLA1* promoter fragment, located at −1989 to −1980 and −1846 to −1840 bp (Figure 6A). Under non‐selective conditions, all yeast transformants showed similar growth. On medium containing 300 ng ml^−1^ aureobasidin A (AbA), yeast co‐transformed with pGADT7‐RcPDF2 and pAbAi‐*RcEXLA1*pro showed stronger growth than the control combinations and remained visible at higher dilutions (Figure 6A). This result indicated that RcPDF2 could bind to the tested *RcEXLA1* promoter fragment in yeast. An HD‐ZIP‐binding element was also found in the *RcEXPA1* promoter at −1054 to −1050 bp. However, no clear difference in yeast growth was observed between pGADT7‐RcHDG2 + pAbAi‐*RcEXPA1*pro and the corresponding controls on medium containing 300 ng ml^−1^ AbA (Figure 6B). The effects of RcPDF2 and RcHDG2 on promoter activity were then examined using DLR assays in *Nicotiana benthamiana* leaves. Co‐expression of 35S::RcPDF2 with *RcEXLA1*pro::LUC produced a stronger luminescence signal and a significantly higher LUC/REN ratio than the control combinations (Figure 6C), indicating that RcPDF2 increased *RcEXLA1* promoter activity. In contrast, co‐expression of 35S::RcHDG2 with *RcEXPA1*pro::LUC did not significantly change the LUC/REN ratio (Figure 6D), which was consistent with the Y1H result.

**Figure 6:**
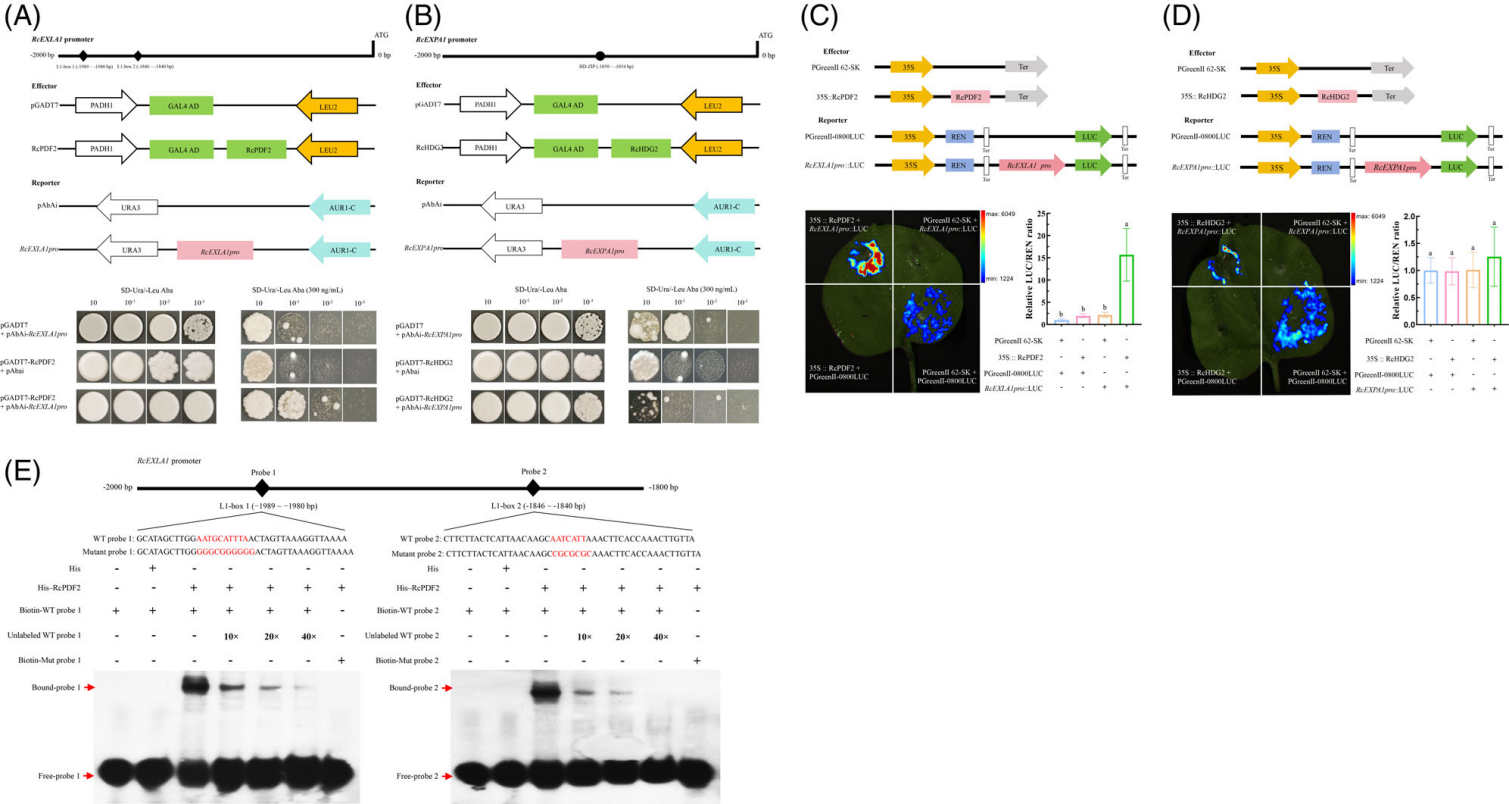
RcPDF2 directly binds to and activates the *RcEXLA1* promoter. (A) Yeast one‐hybrid (Y1H) assay examining the interaction between RcPDF2 and the *RcEXLA1* promoter. (B) Y1H assay examining the interaction between RcHDG2 and the *RcEXPA1* promoter. (C) DLR assay showing the transcriptional activation of the *RcEXLA1* promoter by RcPDF2. Schematic diagrams of the effector and reporter constructs, representative luminescence images, and relative LUC/REN ratios are shown. (D) DLR assay examining the effect of RcHDG2 on *RcEXPA1* promoter activity. (E) Electrophoretic mobility shift assay (EMSA) showing the direct binding of His‐RcPDF2 to two L1‐box elements in the *RcEXLA1* promoter. Values in (C) and (D) represent the means ± SD of three biological replicates. Different letters indicate significant differences according to one‐way anova followed by Tukey's test (*P* < 0.05).

Electrophoretic mobility shift assay (EMSA) was further used to test whether RcPDF2 directly binds to the two L1‐box elements in the *RcEXLA1* promoter. Mobility shifted bands were detected when purified His‐RcPDF2 was incubated with either wild‐type probe containing L1‐box 1, whereas no shifted band was detected with the His‐tag control (Figure 6E). The shifted bands gradually weakened after addition of 10‐, 20‐, and 40‐fold excess unlabeled competitor probes. Mutation of the L1‐box sequences abolished the shifted bands. These results showed that RcPDF2 specifically bound to both L1‐box elements in the *RcEXLA1* promoter. Taken together, the Y1H, DLR, and EMSA results supported the RcPDF2‐*RcEXLA1* relationship, whereas the predicted RcHDG2‐*RcEXPA1* relationship was not supported by the Y1H or DLR assays under the conditions used here.

## DISCUSSION

### Seed enlargement in autotetraploid castor bean is driven by seed coat cell expansion

The size of organs in higher plants is ultimately controlled by the coordinated regulation of cell division and cell expansion (Gonzalez et al., 2012; Tsukaya, 2008). Both processes are regulated in a complex spatial and temporal manner and exhibit a dosage compensation effect, where an increase in cell size is balanced by a corresponding decrease in cell number (Disch et al., 2006). However, the patterns of cell proliferation and expansion in polyploid plants differ markedly from those in diploids. Genome doubling often alters the relative contributions of these two processes, with organ enlargement in polyploids frequently resulting from increased cell size rather than a sustained increase in cell number, although the magnitude and developmental timing of this response vary among organs and species (Corneillie et al., 2019; Li et al., 2012). The seed coat develops from the maternal integuments and forms the outer boundary of the developing seed, thereby potentially constraining the expansion of the endosperm and embryo (Radchuk & Borisjuk, 2014). Given that the seed coat physically constrains the final size of castor bean seeds (Yu et al., 2019), we hypothesized that the larger seeds observed in autotetraploid castor bean plants might be due to an increase in the size and/or number of seed coat cells. To test this hypothesis, we analyzed seed coat sections and found that the volume of seed coat cells was significantly larger in autotetraploids, especially at 35 DAP. Conversely, the number of cells was significantly higher in tetraploids only at 10 DAP, and this increase was temporary. These results clearly indicate that the larger seed size in autotetraploid castor beans is mainly due to the expansion of seed coat cell area.

### Stage‐specific and non‐linear transcriptional responses to genome doubling in autotetraploid seed coats

WGD greatly enhances genomic plasticity, facilitating functional differentiation of duplicated genes, genomic restructuring, and transcriptome changes, thereby contributing to evolution (Comai, 2005; Otto, 2007). In theory, autopolyploidization through somatic chromosome doubling (e.g. by colchicine treatment) should result in a complete doubling of every nuclear chromosome, and thus all genomic regions are expected to be present in four copies. However, in practice, small‐scale copy‐number variations (CNVs), aneuploidy, or gene losses can occur during the process of genome doubling and subsequent propagation, leading to deviations from a strict 4:1 ratio for some loci (Gao et al., 2016; Zhang et al., 2025). Besides, autotetraploids have four sets of homologous chromosomes, so meiotic pairing can involve multivalents, and lead to homologous chromosome exchanges (HEs). These exchanges can result in loss of heterozygosity (LOH), gene conversion, or altered allelic configurations, which in turn may affect gene expression through changes in *cis*‐regulatory elements or epigenetic marks (Janko et al., 2021; Pelé et al., 2018; Seear et al., 2020). Consequently, genome doubling is accompanied by widespread non‐linear changes in gene expression (Chen, 2007; Garcia‐Lozano et al., 2021; Song et al., 2020; Zhang et al., 2015). In tetraploid castor bean seed coats, only a small proportion of genes exhibited a strict 1:1 dosage effect across development, whereas most deviated from proportional expression scaling after WGD. Similar non‐linear dosage responses have also been reported in other autopolyploid species, including Arabidopsis and watermelon, suggesting that transcriptional rebalancing is a common consequence of genome duplication rather than a simple doubling of transcript abundance (Garcia‐Lozano et al., 2021; Song et al., 2020).

Notably, the dosage‐response patterns changed during seed coat development. Early in development, dosage compensation and partial compensation predominated. With development, however, the proportions of genes in the two dosage‐response classes (negative dosage effect and >1:1 dosage effect) increased substantially. At 55 DAP, the proportions of genes showing a negative dosage effect (25.3%) and a >1:1 dosage response (25.8%) reached their highest level across the three developmental stages, and this stage also showed the largest difference in transcript/gene ratios between diploid and tetraploid seed coats. Thus, the tetraploid transcriptome did not shift progressively toward either global activation or repression. Instead, it became increasingly polarized, with expanding subsets of genes showing either marked attenuation or expression beyond the proportional expectation. This stage‐dependent response is consistent with observations in autotetraploid Arabidopsis, in which genome duplication altered selected sets of genes rather than producing uniform genome‐wide transcriptional amplification (Yu et al., 2010). These findings indicate that the transcriptional consequences of genome doubling were strongly shaped by developmental context (Chen, 2007; Comai, 2005), and that this stage‐specific reconfiguration may provide a molecular basis for the activation of cell wall‐related pathways associated with seed coat cell expansion.

### Cell wall loosening facilitates seed coat cell expansion in autotetraploid

Seed size is determined through coordinated growth of the embryo, endosperm, and seed coat. Unlike the embryo and endosperm, the seed coat is derived entirely from the maternal ovule integuments and forms both a physical boundary and a metabolic interface between the mother plant and the developing filial tissues (Ohto et al., 2009; Radchuk & Borisjuk, 2014). Genetic studies in *A. thaliana* have demonstrated that integument cell proliferation and expansion can exert maternal control over final seed size. Both increased cell proliferation and cell expansion can therefore contribute to maternal control of seed size (Garcia et al., 2005; Ohto et al., 2009; Schruff et al., 2006).

Expansins are wall loosening proteins involved in cell expansion and other developmental processes associated with cell wall modification (Cosgrove, 2000; Sampedro & Cosgrove, 2005). Consistent with this role, up‐regulation of expansin genes has been associated with increased cell size in autopolyploid kiwifruit leaves (Zhu et al., 2021). Recent work has further shown that *EXLA1* can directly remodel the cell wall and enhance wall extensibility (Men et al., 2026). In our study, several expansin‐related genes, including *RcEXPA1*, *RcEXPA5*, *RcEXPA9*, *RcEXPA12*, *RcEXPA15*, and *RcEXLA1*, were up‐regulated in tetraploid seed coats, with *RcEXLA1* showing significant up‐regulation at 35 DAP. These expression patterns suggest that expansin mediated wall loosening may contribute to seed coat cell expansion during the active enlargement stage. In addition to wall loosening proteins, factors associated with cell wall structural maintenance may also be involved in this process. EXTs and Extensin‐like proteins (ELPs) are involved in cell wall formation and remodeling (Chakraborty et al., 2015; Soleimani & Alizadeh, 2024; Xu et al., 2008). In our study, *RcEXT3* and *RcELP4* were also up‐regulated in tetraploid seed coats at 35 and 55 DAP, respectively. These results suggest that autotetraploid seed coats may enhance wall extensibility while maintaining structural support during expansion.

### Cell wall remodeling facilitates seed coat cell expansion in autotetraploid

Seed coat expansion also requires coordinated remodeling of the cell wall. Modification of cellulose and hemicellulose networks and the activity of cell wall loosening proteins permit turgor‐driven cell expansion, whereas SCW deposition and lignification can progressively stiffen the tissue and restrict further growth during maturation (Cosgrove, 2005; Di Marzo et al., 2022). In *A. thaliana*, *CESA4*, *CESA7*, and *CESA8* are core components of the SCW cellulose synthase complex (Taylor et al., 2003), and similar roles for these genes have also been reported in other species such as cotton (Zhang et al., 2021), suggesting that this regulatory mechanism is evolutionarily conserved among species. In addition, *XYL1*, *XYL4*, and *XTH* family genes participate in xyloglucan metabolism and cell wall remodeling, thereby influencing wall mechanics and extensibility (Cosgrove, 2005; Di Marzo et al., 2022; Günl & Pauly, 2011; Sechet et al., 2016). In our study, we found significant up‐regulation of *RcCESA4*, *RcCESA7*, *RcCESA8*, *RcXYL1*, *RcXYL4*, *RcIRX9*, *RcXTH15*, *RcXTH27*, and *RcXTH30* in tetraploid seed coats at 10 DAP. These changes suggest that cellulose and hemicellulose‐related remodeling begins early during development. Increased expression of cellulose synthesis genes may help maintain wall support during cell enlargement, whereas higher expression of *XYL1* and XTH genes may favor continued wall remodeling. This early transcriptional activation may contribute to the subsequent expansion of tetraploid seed coat cells.

Lignin is a major strengthening component of SCWs (Büyükkartal et al., 2013), and reduced lignin accumulation after WGD has been reported in several autopolyploid plants, including *A. thaliana* (Corneillie et al., 2019), *Salix* (Serapiglia et al., 2015), and *Populus hopeiensis* (Wu et al., 2023). Consistent with these findings, our results showed that the lignin content in the seed coat of tetraploid castor beans is significantly reduced compared to diploids. Moreover, many lignin biosynthesis‐related genes, including *RcPAL1*, *RcCOMT1*, *Rc4CL2*, *RcCSE*, *RcC4H*, *RcCCR*, *RcCAD*, *RcCCoAOMT1*, and *RcHCT*, were significantly down‐regulated in tetraploid seed coats at 10 and 35 DAP, with the strongest reduction observed at 35 DAP. These patterns suggest that reduced lignin biosynthesis during the early and middle stages may decrease wall rigidity and thereby alleviate mechanical constraints on cell expansion. Together with cellulose‐ and hemicellulose‐related remodeling, reduced lignification may facilitate enlargement of seed coat cells in autotetraploid castor bean.

Together, these data support a sequential cell wall program comprising early establishment of a cellulose and xylan containing framework, selective remodeling by expansin and xyloglucan modifying proteins, concurrent maintenance of wall integrity by *EXT/ELP* proteins, and attenuation of lignification during middle development.

### Stage‐specific IAA, GA and BR responses accompany enhanced seed coat expansion

Auxin is extensively involved in various phases of plant cell growth and development. In Arabidopsis, IAA produced in the endosperm after fertilization is transported to the integuments, where it initiates seed coat development (Figueiredo et al., 2016). A previous study has confirmed that within the Aux/IAA family, reducing the expression of *Sl‐IAA17* in transgenic tomato plants can stimulate endoreduplication in pericarp cells, which enhances cell expansion and ultimately results in larger fruit (Su et al., 2014). We found that higher IAA concentrations in the seed coats of tetraploids at 35 DAP were accompanied by reduced expression of multiple Aux/IAA genes (*RcIAA3*, *RcIAA13*, *RcIAA16*, *RcIAA26*, and *RcIAA27*), whereas *RcGH3.6* and *RcGH3.11* were up‐regulated. We speculate that the down‐regulation of these negative regulatory genes may relieve the inhibition of cell expansion, thereby promoting the expansion of seed coat cells in tetraploid castor bean.

BR also contributes to seed growth through both maternal and zygotic tissues. Previous studies have shown that BR‐deficient Arabidopsis mutants produce smaller seeds, partly owing to reduced integument cell length (Jiang et al., 2013). However, recent evidence indicates that seed coat derived BR signaling regulates cell wall‐related processes and coordinates endosperm proliferation with seed coat expansion (Lima et al., 2024). In this study, the early increase in BR concentration coincided with increased expression of an *RcBSK6* homolog. BSK proteins act downstream of the *RcBRI1* receptor to transmit BR signals (Tang et al., 2008), making this change consistent with altered early BR responsiveness. By contrast, *RcBSK2*, *RcBSK3*, and *RcBZR1* transcripts were reduced at 35 DAP, when BR concentrations no longer differed significantly between ploidy levels. These reductions do not necessarily indicate weaker BR signaling because *RcBZR1* stability, localization, and transcriptional activity are extensively regulated by phosphorylation and protein accumulation (He et al., 2002).

GA also exhibited stage‐specific changes during seed coat development. In the GA signaling pathway, the binding of GA to the GID1 receptor stimulates the formation of the GID1‐GA‐DELLA complex, which triggers DELLA protein ubiquitination and proteolysis. DELLAs have been shown to inhibit GA‐promoted growth by interacting with key regulatory proteins and blocking their DNA‐binding or transactivation activities (Huang et al., 2015). Recent evidence demonstrates that DELLA proteins positively regulate seed size through maternal control in Arabidopsis: the gain‐of‐function *della* mutant *gai‐1* produces larger seeds as a result of increased cell proliferation in ovule integuments (Gomez et al., 2023). In our tetraploid seed coats, the reduced *RcRGL2* expression at 35 DAP may therefore reflect a developmentally regulated reduction in DELLA activity that facilitates cell expansion, consistent with the established role of GA signaling in promoting cell proliferation and expansion in seed tissues.

Notably, hormone signaling pathways do not operate in isolation. In the IAA and BR signaling pathway, RcARF6, RcBZR1, and RcPIF4 can cooperatively regulate a shared set of cell elongation genes (Nemhauser et al., 2004). These three TFs interact at the protein level and cooperatively bind to and regulate common target genes involved in cell expansion. DELLA proteins serve as central integrators of hormonal signals by physically interacting with RcARF6, RcBZR1, and RcPIF4 and inhibiting their transcriptional activity (Oh et al., 2014). Specifically, the DELLA protein can interact with ARF6 and block its DNA‐binding, while DELLAs also interact with and inactivate BZR1 and PIF4. GA‐induced degradation of DELLAs releases this inhibition, thereby strengthening auxin and BR‐mediated activation of cell elongation genes (Bai et al., 2012; Oh et al., 2014). We therefore propose that genome doubling was associated with a more growth permissive hormonal state during early to middle seed coat development. Through partially overlapping IAA, GA, and BR responses, this state could reinforce the expression of expansin, expansin‐like, and XTH genes and thereby support cell wall loosening and cell expansion.

### The RcPDF2‐ *RcEXLA1* regulatory relationship in tetraploid seed coat cell development

HD‐Zip IV TFs, which bind to the L1‐box *cis*‐element, regulate epidermal development by controlling the proliferation and differentiation of epidermal cells during plant growth (Nakamura et al., 2006). In *A. thaliana*, *ATML1*, a homolog of *PDF2*, binds L1‐box *cis*‐elements and has overlapping functions in maintaining shoot epidermal identity (Abe et al., 2003; Nakamura et al., 2006). A related HD‐ZIP IV factor in cotton, GhHD‐1, is preferentially expressed in the ovule epidermis and affects the initiation and early development of seed coat‐derived fibers (Walford et al., 2012). In this study, temporal expression clustering and co‐expression analysis identified RcPDF2 and RcHDG2, both belonging to the HD‐Zip IV subfamily, as candidate regulators of seed coat development. Y1H, EMSA, and DLR assays collectively demonstrated that RcPDF2 directly binds to the L1‐box elements of the *RcEXLA1* promoter and activates its transcription. *RcEXLA1* was preferentially expressed in tetraploid seed coats during early to middle development, coinciding with the period of enhanced cell expansion. Given that *EXLA* proteins preferentially bind pectic polysaccharides and that recombinant *EXLA1* promotes cell wall extension under acidic conditions *in vitro* (Men et al., 2026). RcPDF2‐mediated activation of *RcEXLA1* likely contributes to the wall extensibility required for seed coat cell expansion. Regrettably, the predicted interaction between RcHDG2 and *RcEXPA1* was not supported by our experimental results. AtHDG2 has been reported to participate in epidermal and stomatal development (Peterson et al., 2013). Therefore, the role of RcHDG2 in seed coat development remains to be elucidated.

Moreover, DELLA proteins such as RGL2 have been reported to directly interact with ATML1/PDF2 in Arabidopsis, activating downstream cell wall loosening genes like *EXP8* and promoting epidermal cell elongation (Rombolá‐Caldentey et al., 2014). In this study, tetraploid seed coats showed higher GA levels together with reduced RcRGL2 expression at 35 DAP. This pattern suggests a possible link between GA signaling and RcPDF2, and further functional validation is needed.

## CONCLUSIONS

In this study, we found that the seeds size (including seed length, width, thickness and hundred‐seed weight) of autotetraploid castor beans were significantly larger than that of diploids. Histological analysis further revealed that the enlarged seeds were mainly associated with sustained seed coat cell expansion rather than prolonged cell proliferation. Furthermore, mature tetraploid seed coats contained more cellulose but less hemicellulose and lignin than diploids. At the transcriptome level, most genes in tetraploid seed coats exhibited expression levels that did not follow the expected 1:1 dosage ratio, and the relative dosage responses changed with seed coat development, indicating stage‐specific and non‐linear transcriptional responses following whole‐genome doubling. Transcriptome analysis identified a total of 6043 non‐redundant DEGs between tetraploid and diploid seed coats. The expression of several key genes related to cell expansion and cell wall loosening (e.g. *RcEXPA1*/*5*/*9*/*12*/*15* and *RcEXLA1*, *RcEXTs*, *RcELP*, *RcCESA4/7/8*, *RcXYL1*, and *RcXTH15*/*27*/*30*) was significantly up‐regulated in tetraploids, resulting in enlarged tetraploid seed coats. The expression of lignin synthase genes (*RcPAL1*, *RcCOMT1*, *Rc4CL2*, *RcCSE*, *RcC4H*, *RcCCRs*, *RcCADs*, *RcCCoAOMT1*, and *RcHCT*) was significantly down‐regulated in tetraploids, facilitating seed coat cell enlargement. The stage‐specific DEGs in the IAA, GA, and BR signaling pathways (*RcIAA3*/*13*/*16*/*26*/*27*, *RcRGL2*, and *RcBSK6*) may enhance the expression of genes related to cell enlargement and elongation, thereby promoting seed enlargement in tetraploid castor bean. Importantly, co‐expression analysis together with Y1H, EMSA, and DLR assays showed that RcPDF2 bound to two L1‐box elements in the *RcEXLA1* promoter and increased its promoter activity, suggesting that RcPDF2 contributes to seed size determination by modulating seed coat cell expansion. These coordinated transcriptional changes collectively contribute to seed coat expansion and seed enlargement in autotetraploid castor bean (Figure 7). This study provides new insights into the molecular mechanisms underlying seed enlargement in autopolyploid plants and lays a theoretical foundation for enhancing castor bean yield through polyploid breeding.

**Figure 7:**
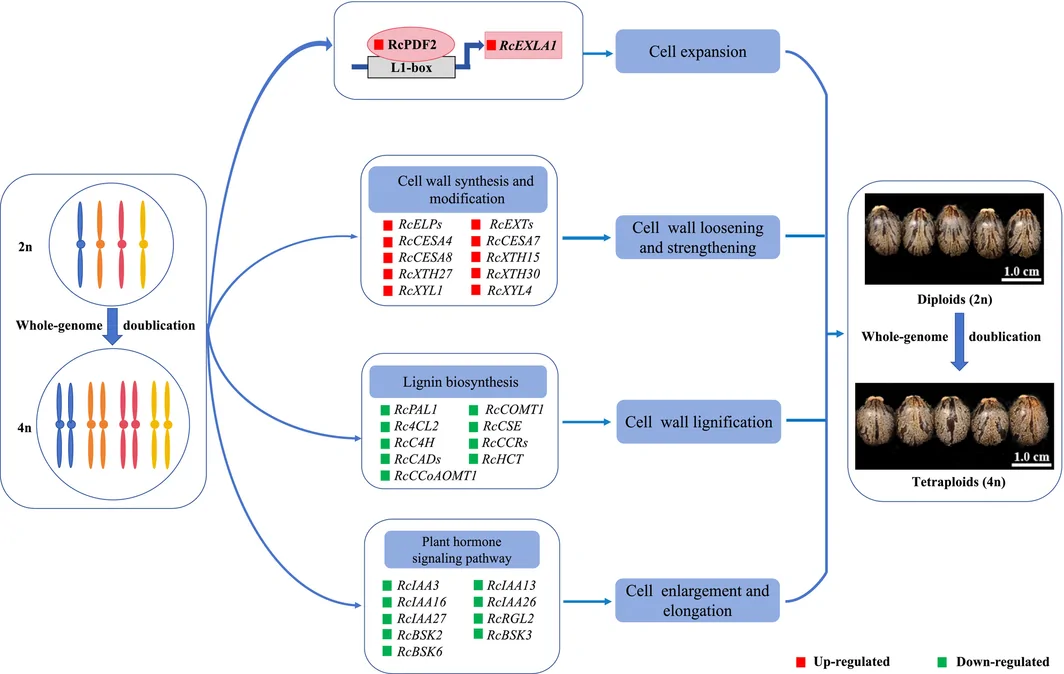
Putative model of the internal mechanisms of bigger seed formation in castor bean after chromosomal doubling. Red and green colors indicate up‐regulated and down‐regulated genes, respectively.

## MATERIALS AND METHODS

### Plant materials, growth conditions, and sample collection

Seeds of diploid castor bean cultivar ZB306 and its synthetic autotetraploid counterpart were obtained from the Germplasm Bank of Wild Species, Kunming Institute of Botany, Chinese Academy of Sciences, Kunming, China. After germination on moist filter paper for 2 days, seedlings were planted in plastic pots containing a mixture of turfy soil, perlite, and sand (1:1:2). The plants were grown in a greenhouse at Southwest Forestry University, Kunming, China. Flowers were tagged on the day of pollination, which was defined as 0 DAP. Developing seeds were collected from diploid and tetraploid plants at 10, 35, and 55 DAP, and seed coat tissues were carefully separated from the embryos and endosperm. Isolated seed coats were stored at −80°C for plant hormone quantification, RNA sequencing, and qRT‐PCR validation. For histological analysis, the remaining seeds and isolated seed coats were also fixed in formalin‐acetic acid‐alcohol (FAA) solution (5% formaldehyde, 5% acetic acid, and 90% ethanol) for at least 48 h. Three biological replicates were used for all experiments.

### Phenotypic, histological and cytological analysis

Mature seeds were independently harvested from diploid and autotetraploid plants. Seed length, width, and thickness were measured using a digital Vernier caliper. Seed length was measured along the longitudinal axis, seed width at the widest position perpendicular to the longitudinal axis, and seed thickness as the maximum distance between the two lateral surfaces. Hundred‐seed weight was determined using an electronic balance with a precision of 0.0001 g; 100 seeds were measured for each ploidy level. Three independent biological replicates were analyzed for all mature seed traits.

Fresh seed coats at 10 and 35 DAP were analyzed by paraffin sectioning to examine cytological characteristics, while dry seed coats at 55 DAP were observed using field‐emission scanning electron microscopy (since paraffin sectioning was not feasible for the 55 day dry seed coats). Fresh seed coats were fixed in FAA for at least 48 h, followed by dehydration (from 70, 80, 90, 95 to 100%). Tissue clearing was performed by sequential immersion in an ethanol: xylene solution (1:1, v/v) for 1.5 h, followed by three 1.5 h cycles in 100% xylene. The specimens were subsequently embedded in paraffin wax at 65°C for 15 h; paraffin sections (4–6 μm slices) were prepared with a rotary microtome and stained with Safranine O‐Fast Green. After staining, the sections were mounted with neutral balsam, air‐dried at 37°C in an incubator, and imaged under a Nikon Eclipse Ci microscope (Tokyo, Japan).

Give that seed coats at 55 DAP were unsuitable for paraffin sectioning, epidermal cell morphology was examined using field‐emission scanning electron microscopy. Seed coats were placed in cryotubes, snap‐frozen in liquid nitrogen for 0.5–2 h, and subsequently lyophilized for 2 days using a freezer dryer (Model DC401, Yamato Scientific Co., Ltd., Tokyo, Japan). Dried samples were gold‐coated for 30 sec with an ion sputter coater (MC1000, Hitachi High‐Technologies Corporation, Tokyo, Japan) and subsequently observed using a FE‐SEM (SU8010, Hitachi High‐Technologies Corporation) operated at an accelerating voltage of 3 kV. The cell size, cell number, and seed coat thickness were determined using ImageJ software, with 30 randomly selected visual fields analyzed per biological replicate for statistical validity.

Seed coat cell area, cell density, cell number, and seed coat thickness were measured from transverse and longitudinal sections using ImageJ v1.52v. For each biological replicate, 30 randomly selected microscopic fields were analyzed. Seed coat thickness was measured as the perpendicular distance between the outer and inner boundaries of the seed coat at six positions in each section, and the mean value from six sections was used for each biological replicate. Seed coat cell density was calculated as the number of cells per unit area in transverse and longitudinal sections. The total number of seed coat cells was estimated by multiplying cell density by the corresponding seed coat area.

### Measurement of seed coat lignin, cellulose, hemicellulose and plant hormone contents

Mature seed coats were isolated from dry seeds of diploid and tetraploid castor bean for lignin, cellulose, hemicellulose determination. Lignin content was measured following the standard method provided by the National Renewable Energy Laboratory (NREL, Golden, CO, USA) (Sluiter et al., 2008). Briefly, 0.3 g of dried seed coat powder was hydrolyzed with 72% sulfuric acid at 30°C for 1 h. The hydrolysate was then diluted and hydrolyzed again, after which the acid‐insoluble residue was collected, dried, and weighed. Lignin content was calculated as a percentage of the initial sample dry weight. Cellulose content was measured by the sulfuric acid‐anthrone method. Samples were digested with 80% H_2_SO_4_ and reacted with 2% anthrone and concentrated H_2_SO_4_. Absorbance was measured at 620 nm using purified cellulose as the standard. Hemicellulose content was measured by HCl hydrolysis and the 3,5‐dinitrosalicylic acid method. Samples were treated with 80% calcium nitrate and hydrolyzed with 2 mol L^−1^ HCl in a boiling water bath for 45 min. Absorbance was measured at 540 nm using glucose as the standard, and hemicellulose content was calculated from the reducing sugar content with a conversion factor of 0.9. Cell wall component contents were expressed on a dry weight basis. Three independent biological replicates were analyzed for each ploidy level.

For plant hormone analysis, seed coat tissues were collected from developing seeds at 10, 35, and 55 DAP. The content of auxin (IAA; catalog no. YJ147100), GAs (YJ062451), BRs (YJ036309), and CTKs (YJ020295) was measured using enzyme‐linked immunosorbent assays (ELISA) kits (Shanghai, China) according to the manufacturer's instructions. Three independent biological replicates were analyzed for each ploidy stage combination.

### RNA sequencing, transcript assembly, and expression analysis

Seed coats from diploid and tetraploid castor bean plants at 10, 35, and 55 DAP were used for RNA‐seq. Diploid samples were designated D1, D3, and D5, whereas tetraploid samples were designated T1, T3, and T5. Total RNA was extracted using the EASYspin Plant microRNA Kit (Aidlab Biotechnologies Co., Ltd., Beijing, China) according to the manufacturer's instructions. RNA quality was examined with a NanoDrop2000 spectrophotometer (Thermo Fisher Scientific, Waltham, MA, USA). Poly(A) containing transcripts were purified from total RNA using oligo(dT) magnetic beads and then reverse‐transcribed into cDNA for library construction. Qualified libraries were sequenced on the Illumina NovaSeq 2000 platform by Wuhan Frasergen Bioinformatics Co., Ltd. (Wuhan, China). Raw reads were quality filtered using fastp v0.23.4 (Chen et al., 2018), and the clean reads were aligned to the *R. communis* ZB306 reference genome obtained from EupDB (http://eupdb.liu‐lab.com/download/; Liu et al., 2024) using HISAT2 v2.0.4 (Kim et al., 2015). Gene expression level was quantified with featureCounts v2.0.2 (Liao et al., 2014). Raw counts were used for differential expression analysis, whereas FPKM and TPM values were used for expression visualization and relative dosage‐response analysis, respectively.

Transcripts were assembled for each sample using StringTie v2.1.1 with the ZB306 genome annotation as a guide (Pertea et al., 2015). Expressed genes and transcripts were counted for each biological replicate and averaged for each ploidy and stage combination. Relative dosage responses were analyzed separately at each developmental stage using genes with TPM >0.01 in both ploidy levels. The ploidy adjusted ratio was calculated as:

$$R=\left(T/4\right)/\left(D/2\right)=T/\left(2D\right),$$

where *T* and *D* are the mean TPM values of tetraploid and diploid seed coats, respectively. Genes were divided into five groups: negative dosage effect (*R* < 0.25), dosage compensation (0.25 ≤ *R* < 0.50), partial dosage compensation (0.50 ≤ *R* < 0.75), 1:1 dosage effect (0.75 ≤ *R* ≤ 1.00), and >1:1 dosage effect (*R* > 1.00) (Garcia‐Lozano et al., 2021). As RNA spike‐ins and total RNA measurements per cell or genome were not available, these groups represent relative TPM based dosage responses rather than absolute transcript abundance.

Differential gene expression analysis was performed using DESeq2 in R by comparing tetraploid and diploid seed coats separately at 10, 35, and 55 DAP. Genes with a false discovery rate (FDR) <0.05 and |log2FC| >0.75 were identified as DEGs. Gene ontology (GO) functional enrichment analysis and KEGG pathway enrichment analysis were performed using the OmicShare tools platform (https://www.omicshare.com/tools/), and the top 15 KEGG pathways were selected for visualization. Heatmaps were generated using mean FPKM values of three biological replicates after row‐wise *Z*‐score normalization (https://www.omicshare.com/tools/). All DEGs from the three developmental stages were grouped into five temporal expression clusters using Mfuzz based on *Z*‐score normalized mean FPKM values (https://www.bioinformatics.com.cn).

### TF identification, co‐expression analysis and candidate gene selection

TFs from the castor bean protein were identified used PlantTFDB (https://planttfdb.gao‐lab.org/prediction.php), and putative Arabidopsis homologs were assigned by BLASTP against the *A. thaliana* protein database (https://www.arabidopsis.org/). TFs with assigned *A. thaliana* homologs were grouped into five clusters based on their *Z*‐score‐normalized mean FPKM values. Subsequently, co‐expression analysis was performed between TFs and seed coat development‐related genes across the 18 RNA‐seq samples using Pearson correlation analysis. Gene pairs with |*r*| ≥ 0.8 and *P* < 0.05 were retained, and the network was visualized using Cytoscape v3.10.1 (Shannon et al., 2003). Candidate TF‐gene pairs were selected based on their expression patterns, correlations, functional annotations, and predicted promoter *cis*‐elements.

The 2 kb upstream promoter sequences of *RcEXLA1* and *RcEXPA1* were retrieved from the castor bean ZB306 genome using TBtools, and *cis*‐elements were predicted using PLACE (http://www.dna.affrc.go.jp/PLACE/). Two L1‐box elements at −1989 to −1980 and −1846 to −1840 bp in the *RcEXLA1* promoter and one predicted HD‐ZIP‐binding site at −1054 to −1050 bp in the *RcEXPA1* promoter were selected for subsequent binding assays.

### Yeast one‐hybrid assays and dual‐luciferase reporter assay

Y1H assays were used to test the binding of RcPDF2 and RcHDG2 to the *RcEXLA1* and *RcEXPA1* promoters. The *RcEXLA1* promoter fragment (−2000 to −1735 bp) containing two L1‐box elements and the *RcEXPA1* promoter fragment (−1206 to −935 bp) containing the predicted HD‐ZIP‐binding site were cloned into pAbAi. The full‐length coding sequences of RcPDF2 and RcHDG2 were cloned into pGADT7. The bait and prey constructs were transformed into the Y1H Gold yeast strain according to the manufacturer's protocol (Coolaber, Beijing, China). Transformants were grown on SD/−Ura/−Leu medium containing 0, 100, 300, 500, or 700 ng ml^−1^ AbA at 30°C for 3 days. Primer sequences are listed in Table S1.

For DLR assays, the *RcEXLA1* and *RcEXPA1* promoter fragments were cloned into pGreenII 0800‐LUC, and the coding sequences of RcPDF2 and RcHDG2 were cloned into pGreenII 62‐SK. Reporter and effector constructs, together with the corresponding empty vector controls, were introduced into *Agrobacterium tumefaciens* GV3101 and co‐infiltrated into *N. benthamiana* leaves. LUC signals were detected using a Tanon 5200 imaging system (Tanon, Shanghai, China) after application of 1 mM d‐luciferin. LUC and REN activities were measured using a Dual‐Luciferase Reporter Assay Kit, and relative promoter activity was calculated as the LUC/REN ratio. Three biological replicates were used for each assay. Primer sequences are listed in Table S1.

### Electrophoretic mobility shift assay

The RcPDF2 coding sequence was cloned into pET‐28a(+) and expressed as a His‐tagged protein in *Escherichia coli* Origami2 (DE3). Protein expression was induced with 0.5 mmol L^−1^ IPTG at 27°C for approximately 8 h, and His‐RcPDF2 was purified using Ni‐NTA affinity chromatography. Protein from the empty pET‐28a(+) vector was used as the His‐tag control. Biotin labeled wild‐type and mutant probes containing the two L1‐box elements in the *RcEXLA1* promoter were used for EMSA. The L1‐box sequences in the mutant probes were replaced with CGCGCGC. Unlabeled wild‐type probes at 10‐, 20‐, and 40‐fold excess were used for competition assays. Protein DNA complexes were separated by native polyacrylamide gel electrophoresis and detected using a streptavidin‐horseradish peroxidase chemiluminescence system. Probe sequences are listed in Table S1.

### Quantitative real‐time PCR

Total RNA was extracted using the RNAprep Pure Plant Plus Kit (Tiangen, Beijing, China), and cDNA was synthesized using the All‐in‐One First‐Strand cDNA Synthesis SuperMix for qPCR (TransGen Biotech, Beijing, China). qRT‐PCR was performed using PerfectStart Green qPCR SuperMix (TransGen Biotech, Beijing, China) on a CFX96 Real‐Time PCR System. Relative expression levels were calculated using the 2^−∆∆Ct^ method with *RcActin* as the internal reference gene. Three biological replicates with three technical replicates each were analyzed. Primer sequences are listed in Table S1.

### Statistical analysis

Data are presented as means ± SD of three biological replicates. Differences between diploid and tetraploid samples were analyzed using Student's *t*‐test. Dual‐luciferase data were analyzed by one‐way anova followed by Tukey's multiple comparison test. Statistical analyses were performed using SPSS v20.0, with *P* < 0.05 considered statistically significant.

## AUTHOR CONTRIBUTIONS

DH: Investigation, data curation, formal analysis, writing—original draft. TY and XC: Data curation, formal analysis. XX: Data curation, formal analysis. CL: Software. AY: Formal analysis. BH: Investigation. AL: Writing, review and editing, conceptualization, funding acquisition, supervision. JW: Conceptualization, formal analysis, funding acquisition, writing, review and editing, supervision. All authors critically revised the manuscript.

## CONFLICT OF INTEREST

The authors declare no conflict of interest.

## Supporting information


**Figure S1.** Changes in endogenous hormone contents in diploid and tetraploid castor bean seed coats during development.
**Figure S2.** RT‐qPCR validation of RNA‐seq expression patterns of selected genes in diploid and tetraploid castor bean seed coats.
**Figure S3.** Overview of gene expression levels and differentially expressed genes in diploid and tetraploid castor bean seed coats.

**Table S1.** Primer sequences used in this study.
**Table S2.** The statistic of RNA‐seq in diploids and tetraploids of *Ricinus communis* L.
**Table S3.** Classification of gene dosage responses in diploid and autotetraploid seed coats at 10 DAP.
**Table S4.** Classification of gene dosage responses in diploid and autotetraploid seed coats at 35 DAP.
**Table S5.** Classification of gene dosage responses in diploid and autotetraploid seed coats at 55 DAP.
**Table S6.** Top 15 KEGG pathways enriched among genes showing 1:1 and >1:1 dosage effects at 10 DAP.
**Table S7.** Top 15 KEGG pathways enriched among genes showing negative dosage effects at 35 DAP.
**Table S8.** Differentially expressed genes between diploid and tetraploid seed coats at 10 DAP.
**Table S9.** Differentially expressed genes between diploid and tetraploid seed coats at 35 DAP.
**Table S10.** Differentially expressed genes between diploid and tetraploid seed coats at 55 DAP.
**Table S11.** Top 15 KEGG pathways enriched among DEGs assigned to Clusters 1–5.
**Table S12.** Genes associated with cell division, cell expansion, and cell wall remodeling.
**Table S13.** Genes associated with cellulose and hemicellulose biosynthesis and lignin biosynthesis.
**Table S14.** Genes associated with IAA, GA, and BR signaling.
**Table S15.** Expression profiles, temporal clustering, differential expression, and functional annotations of 453 transcription factors in developing castor bean seed coats.
**Table S16.** Expression profiles, differential expression statistics, and functional annotations of 30 TFs and 20 seed coat development‐related genes included in the co‐expression network.

## Data Availability

The raw sequence data reported in this paper have been deposited in the Genome Sequence Archive in the National Genomics Data Center, China National Center for Bioinformation/Beijing Institute of Genomics, Chinese Academy of Sciences (GSA: CRA037205) that are publicly accessible at https://ngdc.cncb.ac.cn/gsa.
