# Supplementary material for: Molecular mechanisms underlying seed enlargement mediated by seed coat development in autotetraploid castor bean
Source: Plant J. 2026 Sep 28;127(6):e71146. doi: 10.1111/tpj.71146 (PMC13618894; doi:10.1111/tpj.71146)
Supplement: Supplementary file 1 [file TPJ-127-0-s001.docx]

**Supplementary Figures:**

Figure S1 Changes in endogenous hormone contents in diploid and tetraploid castor bean seed coats during development. Contents of IAA (a), GA (b), BR (c), and CTK (d) in diploid and tetraploid seed coats at 10, 35, and 55 days after pollination (DAP). Data are shown as means ± SD of three independent biological replicates. Asterisks indicate significant differences between diploids and tetraploids at the same developmental stage determined using Student’s *t*-test (* 0.01 < *p* < 0.05, ** 0.001 < *p* < 0.01, *** *p* < 0.001); ns, not significant.

Figure S2 RT-qPCR validation of RNA-seq expression patterns of selected genes in diploid and tetraploid castor bean seed coats. Expression patterns of *PAL1* (Rc10G022158), *CCR1* (Rc07G017020), *C4H* (Rc02G003473), *CYP76C3* (Rc05G010356), *EXPA1* (Rc01G000588), *EXLA1* (Rc07G016088), *PDF2* (Rc05G009388), *MYB61* (Rc10G024480), and *DIR19* (Rc06G014624) were compared between RT-qPCR and RNA-seq analyses. Columns represent relative expression levels determined by RT-qPCR, whereas lines represent RNA-seq expression levels based on FPKM values. Data are shown as means ± SD of three independent biological replicates. D1 and D3 represent diploid seed coats at 10 and 35 days after pollination (DAP), respectively; T1 and T3 represent the corresponding tetraploid seed coats.

Figure S3 Overview of gene expression levels and differentially expressed genes in diploid and tetraploid castor bean seed coats. (a) Distribution of gene expression levels across the 18 RNA-seq samples. Genes were grouped into six expression intervals according to their FPKM values: 0 ≤ FPKM < 1, 1 ≤ FPKM < 5, 5 ≤ FPKM < 10, 10 ≤ FPKM < 30, 30 ≤ FPKM < 50, and FPKM ≥ 50. (b) Numbers of total, up-regulated, and down-regulated differentially expressed genes (DEGs) between tetraploid and diploid seed coats at 10, 35, and 55 days after pollination (DAP). Up- and down-regulation refer to expression in tetraploids relative to diploids. D1, D3, and D5 represent diploid seed coats at 10, 35, and 55 DAP, respectively; T1, T3, and T5 represent the corresponding tetraploid seed coats.
